# PlantRNA_Sniffer: A SVM-Based Workflow to Predict Long Intergenic Non-Coding RNAs in Plants

**DOI:** 10.3390/ncrna3010011

**Published:** 2017-03-04

**Authors:** Lucas Maciel Vieira, Clicia Grativol, Flavia Thiebaut, Thais G. Carvalho, Pablo R. Hardoim, Adriana Hemerly, Sergio Lifschitz, Paulo Cavalcanti Gomes Ferreira, Maria Emilia M. T. Walter

**Affiliations:** 1 Departamento de Ciência da Computação, Universidade de Brasília, Brasília—DF 70910-900, Brasil; maciel.lucas@outlook.com; 2 Laboratório de Química e Função de Proteínas e Peptídeos, Universidade Estadual do Norte Fluminense, Campos dos Goytacazes—RJ 28013-602, Brazil; cgrativol@uenf.br; 3 Instituto de Bioquímica Médica Leopoldo de Meis, Universidade Federal do Rio de Janeiro, Rio de Janeiro—RJ 21941-901, Brazil; flaviabqi@gmail.com (F.T.); thaislouise@hotmail.com (T.G.C.); phardoim@gmail.com (P.R.H.); hemerly@bioqmed.ufrj.br (A.H.); paulof@bioqmed.ufrj.br (P.C.G.F.); 4 Departamento de Informática, Pontifícia Universidade Católica do Rio de Janeiro, Rio de Janeiro—RJ 22451-900, Brazil; sergio@inf.puc-rio.br

**Keywords:** long non-coding RNAs, long intergenic non-coding RNAs, plants, sugarcane, maize, SVM-based workflow, bioinformatics

## Abstract

Non-coding RNAs (ncRNAs) constitute an important set of transcripts produced in the cells of organisms. Among them, there is a large amount of a particular class of long ncRNAs that are difficult to predict, the so-called long intergenic ncRNAs (lincRNAs), which might play essential roles in gene regulation and other cellular processes. Despite the importance of these lincRNAs, there is still a lack of biological knowledge and, currently, the few computational methods considered are so specific that they cannot be successfully applied to other species different from those that they have been originally designed to. Prediction of lncRNAs have been performed with machine learning techniques. Particularly, for lincRNA prediction, supervised learning methods have been explored in recent literature. As far as we know, there are no methods nor workflows specially designed to predict lincRNAs in plants. In this context, this work proposes a workflow to predict lincRNAs on plants, considering a workflow that includes known bioinformatics tools together with machine learning techniques, here a support vector machine (SVM). We discuss two case studies that allowed to identify novel lincRNAs, in sugarcane (*Saccharum* spp.) and in maize (*Zea mays*). From the results, we also could identify differentially-expressed lincRNAs in sugarcane and maize plants submitted to pathogenic and beneficial microorganisms.

## 1. Introduction

The development of new techniques of high-throughput sequencing and the large amount of sequencing projects have been creating enormous volumes of biological data [1], which have been revealing an increasingly number of non-coding RNAs (ncRNAs) in eukaryotic genomes [2]. These ncRNAs directly act in the cellular structures, as well as in catalytic and regulatory processes [3]. Between two distinct ncRNA classifications we have (a) small RNAs, those well-known structured RNAs with lengths between 20 and 30 nucleotides, and (b) long ncRNAs (lncRNAs), those presenting more than 200 nucleotides and a poor capacity to code proteins, which represent the least understood transcripts today [4,5,6].

**Figure 1:**
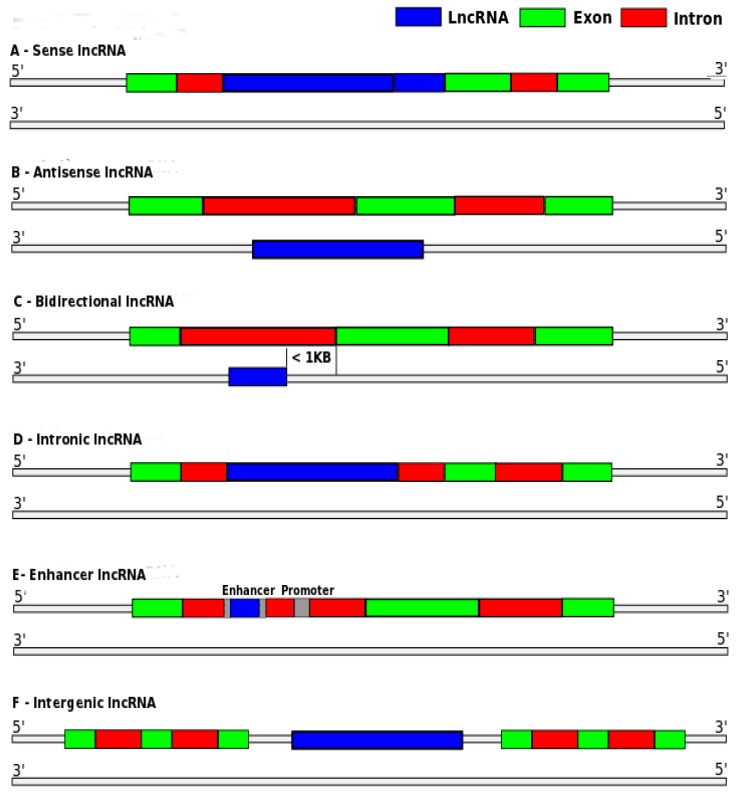
Long non-coding RNA (lncRNA) categories: (**a**) sense; (**b**) antisense; (**c**) bidirectional; (**d**) intronic; (**e**) enhancer; and (**f**) intergenic. Adapted from [7].

lncRNAs may be classified into six major categories: (i) sense or (ii) antisense, when the lncRNA overlaps the transcription region of one or more exons of another gene, on the same or the opposite strand, respectively; (iii) bidirectional, when the start of lncRNA transcription and another gene in the opposite strand are close; (iv) intronic, when the lncRNAs are derived entirely from introns; (v) enhancer, when the lncRNAs are located in enhancer regions; or (vi) intergenic, also called lincRNA, when the lncRNA is located in the interval between two genes [4,7]. Figure 1 illustrates these categories.

To predict ncRNAs and their corresponding genes, as well as to simplify their analyses by avoiding the complications arising from overlap with other types of genes, recent focus has been set to lincRNAs. These lincRNAs derive from genes and, thus, are “genic”, but do not overlap exons from either protein-coding or other non-lincRNA types of genes [8].

In this sense, lncRNAs can be divided in two subsets: lncRNAs that overlap with protein-coding genes; and lincRNAs, found at intergenic regions. The evolutionary history and patterns of conservation (and, thereby, prediction patterns) of these two lncRNA subsets are very different. For instance, lncRNAs that overlap with protein-coding genes look like protein-coding genes. They are spliced (predominantly), exhibit elevated conservation (relative to lincRNAs), and are expressed (typically) in a manner that looks like the protein-coding gene they overlap. Therefore, they are difficult to predict by a machine learning algorithm.

There are some computational methods, based on machine learning techniques, designed to discriminate ncRNAs from protein coding transcripts (PCTs), and to identify some classes of ncRNAs. CONC (coding or non-coding) [9], CPC (coding potential calculator) [10], and PORTRAIT [11] have been developed to discriminate protein-coding genes from ncRNAs. CONC is slow on analyzing large datasets, CPC works well with known protein coding transcripts, but may tend to classify novel PCTs into ncRNAs, if they have not been recorded in the protein databases. PSoL [12], SnoReport [13], RNAsnoop [14], and SnoStrip [15] are methods designed to classify small ncRNAs. LncRScan-SVM [16], lncRNA-MFDL [17], lncRNA-ID [18], lncRNApred [19], PLEK [20], and CNCI [21] are methods that use machine learning techniques in order to classify lncRNAs. In particular, ISeeRNA [22] and linc-SF [23] use machine learning techniques to classify lincRNAs in humans and mice. In plants, there are projects to find and characterize lncRNAs [24,25,26], relying mostly in laboratorial techniques. Wang et al. [24] also identified lincRNAs, using a specific maize assembly.

On the other hand, methods to predict lincRNAs in organisms (plants in specific) have to have a reference genome. Additionally, the available methods (described previously) work well for specific organisms (mainly human and mouse), but in general, do not generalize, i.e., they do not produce good results for species different from the ones they have been designed to. Among the prediction methods, only PLEK [20] and CNCI [21] were trained to discriminate lncRNAs from PCTs in plants, but they are not focused on lincRNAs. As far as we know, there are no methods nor workflows specially designed to predict lincRNAs in plants. 

In this context, this work proposes a workflow that uses machine learning and some bioinformatics tools to predict lincRNAs in plants. This workflow, composed of phases to computationally predict lincRNAs, aims to indicate potential lincRNAs, which should be further studied to find their biological rules, e.g., lincRNA association with diseases. We also discuss two case studies using this workflow, for sugarcane and maize. 

## 2. Methods

### 2.1. Basic Concepts 

#### 2.1.1. Machine Learning

Machine learning, a subarea of artificial intelligence, focuses on the development of algorithms that detect patterns and learn from experience [27]. Very briefly, four paradigms of learning are known: (i) supervised, which seeks to identify features that can be used to classify data in already known classes (labeled), using training datasets for the construction of the model and testing data sets for validation; (ii) unsupervised, which recognizes patterns in data, not previously labeled; (iii) reinforcement, which is based on improving learning taking actions in a particular environment to maximize receiving rewards; and (iv) semi-supervised, which seeks to extend the supervised method with unsupervised learning techniques, to improve the construction of the classification model.

#### 2.1.2. Support Vector Machine

**Figure 2:**
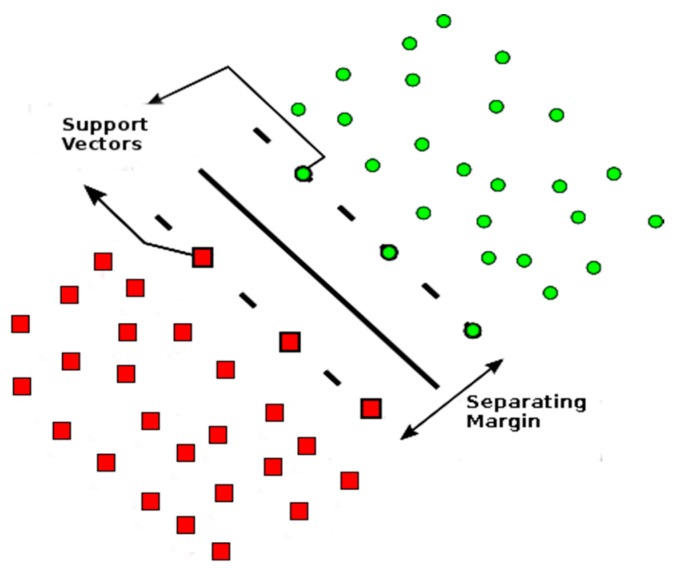
Example of support vectors with dimension 2, where the support vectors separate circles and square objects. Adapted from [30].

A support vector machine (SVM) is a machine learning supervised method, which classifies groups based on the creation of separating margins. These margins, delineated by a fraction of the training data, are called support vectors [28], and separates sets of data into known labeled classes (see Figure 2).

In the training phase, the input data is separated in classes, using a function constructed by the model, called hypothesis. After, the testing phase is executed, in which part of the input data (not used before) is classified according to the model built on the training phase. In this phase, some measures (e.g., accuracy) are used to evaluate the number of input objects classified correctly (according to the labels they should be classified). 

**Figure 3:**
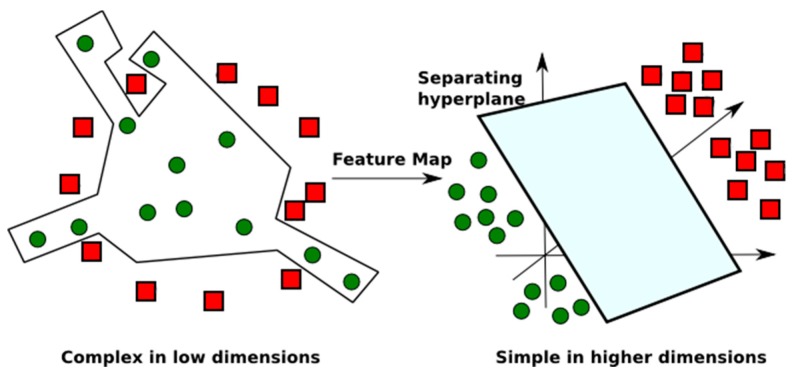
Non-linear separable data in low dimension, mapped to a higher dimension, so that the separation of the groups may be simplified in a hyperplane in a higher dimension. Adapted from [31].

SVM is a non-parametric method that is not limited by the size of the training dataset. Basically, SVM generates models used for classification and regression. In both cases, if SVM is not able to clearly create the margins (the support vectors), it can construct hyperplanes in a high dimensional space, so that it selects the ones with the largest margin, related to the training data [29]. In more details, the SVM model tries to find a linear separator to distinguish the groups of objects of the input dataset. In some cases, the margins cannot be created when simple linear separation methods are used for non-linear separable data. To solve this problem, SVM uses kernel functions to increase the dimension of the space, such that the dataset can be linearly separable at higher dimensions (see Figure 3). This task can reduce overfitting, a phenomenon that occurs when the constructed model fits so well to specific training data that it is not able to reliably predict general untrained data. 

In addition to kernelization, some techniques that improve the model can be used, such as *k*-fold cross-validation and grid search, to optimize the SVM parameters. In *k*-fold cross-validation, the data is first partitioned into *k* equally (or nearly equally) sized segments or folds. Subsequently *k* iterations of training and validation are performed such that within each iteration a different fold of the data is held-out for validation while the remaining *k*−1 folds are used for learning [32]. Alongside *k*-fold cross-validation, we have the SVM kernel parameters, C and gamma, which can affect the capacity of generalization of the model. Thus, they can be optimized using a grid search. This technique changes these two parameters until finding the best ones, those that generate the best model.

In this work, we chose SVM based on: (a) the construction of a maximum separating margin, which lowers the classification errors; (b) the creation of hyperplanes, even in the cases where classes are not linearly separable, using kernels; and (c) the method is non-parametric, thus, it enables a better generalization of the constructed model. This last characteristic (non-parametric) is one of the main motivations to use SVM. We do not limit the volume of data used in the training phase, which is a good aspect, since even if there is not enough data labeled as lincRNAs, it does not affect the construction of a model exhibiting a good prediction. In addition, we can use techniques such as *k*-fold cross-validation and grid search to refine the construction of the predictive model. 

There are several available libraries for SVM, but most of them use the implementation developed by Chang and Lin [33], called libSVM. In our SVM model building, the package e1071 [34] was used, which offers an interface to libSVM.

#### 2.1.3. Training Features

Training features are a key part of the SVM method. They allow to build a correct model, associating specific characteristics to each class, given as input. In our case, the features are biological factors that allow to characterize lincRNAs. 

First, as seen in projects such as CPC [10], homology is an important feature that enables one to classify a transcript as lncRNA. According to iSeeRNA [22], this feature is strongly correlated to the conservation of the transcript. Since these values can be found in the UCSC Genome Browser [35] for some organisms, conservation can be a training feature, when available for the organism of interest. However, they are not available for plants, analyzed in our case studies. 

Since lincRNAs are sequences that have ORFs (open reading frames) but are not expressed into proteins, i.e., they have poor capacity of coding proteins, we chose features related to ORFs as follows: the first one is the proportion between the ORF length divided by the sequence length, which captures the percentage of the coding potential capacity; the other feature is the ORF length, explained by Dinger et al. [36], who defined a lincRNA as a transcript with ORF region length less than 100 amino acids.

Additionally, analyzing all of the 2-, 3-, and 4-nucleotides with principal component analysis (PCA) [37], we have identified the 10 nucleotide pattern frequencies more significant to discriminate lincRNAs and PCTs.

In summary, we used 12 features in our case studies: ORF proportion, ORF length, and 10 nucleotide pattern frequencies. 

### 2.2. Workflow to Predict LincRNAs in Plants

#### 2.2.1. SVM Model to Predict LincRNAs

**Figure 4:**
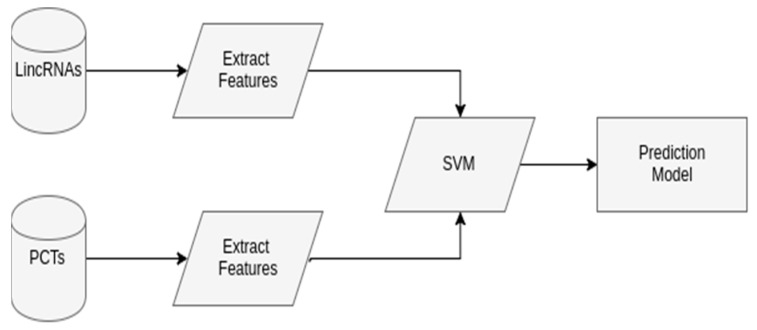
The generic support vector machine (SVM) model to predict long intergenic non-coding RNA (lincRNAs) in plants, with two phases. In Extract features, a given transcript is screened, so that features that characterize the lincRNAs and protein coding transcripts (PCTs) are extracted, in this case, open reading frames (ORF) length, ORF proportion (ORF length divided by the transcript length), and the 10 more significant 2-, 3-, and 4-nucleotides to identify lincRNAs according to principal component analysis (PCA). In SVM, the SVM model is constructed, with balanced data of lincRNAs and PCTs features, 10-fold cross-validation, grid-search, and using 80% of the data as the training set and the other 20% as test. The input data include lincRNAs as the positive set, and PCTs as the negative set. The output is the constructed SVM model to predict lincRNAs.

In this paper, we propose and develop a workflow that considers a SVM model to predict lincRNAs in plants. This SVM model uses two labeled classes, lincRNAs as the positive dataset, and PCTs as the negative dataset. In some cases, the genome of the organism of interest is not known yet. When there are no available lincRNAs nor PCTs, phylogenetically close organisms can be used. Additionally, if data labeled as lincRNAs cannot be found, lncRNAs are used instead, since the workflow has a phase to verify if the transcript is intergenic (i.e., if the lncRNA occurs between two genes coding for proteins). This way, the predicted lncRNAs may be characterized as lincRNAs. Figure 4 shows the generic SVM model. 

#### 2.2.2. Generic Workflow to Predict LincRNAs in Plants

**Figure 5:**
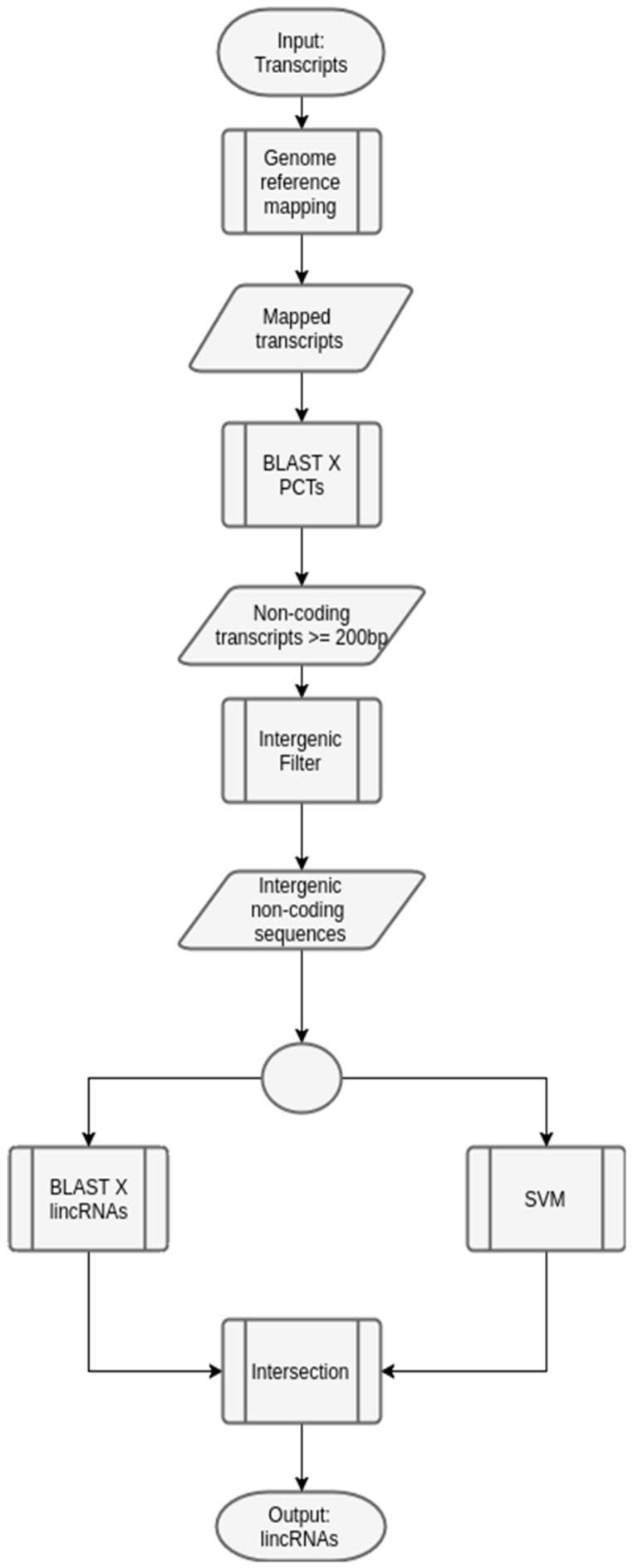
A generic workflow to predict lincRNAs in plants. The input set of transcripts is initially used in a mapping phase, with a reference organism (the same organism of the input transcripts, or another one evolutionarily close). After, BLAST [38] is executed, with PCTs of the same organism used in the previous phase, in order to remove potential coding transcripts, which may not be lincRNAs. Next, only the transcripts with length greater or equal to 200 bp (a key characteristic when predicting lncRNAs) are selected. These transcripts are filtered, according to the mapping step, and only the intergenic ones are selected. Following, they are analyzed by both the specially-constructed SVM model (described before), and BLAST against a database of lincRNAs of the chosen organism. The transcripts that appear both in the set generated by the SVM model and BLAST annotation are the output of the workflow, and constitute potential lincRNAs.

As said before, there are prediction methods for lincRNAs based on machine learning [22], including the one we developed in this project. However, in order to improve the prediction of lincRNAs in plants, we propose a workflow that uses some bioinformatics tools, e.g., mapping and annotation programs, which will interact with machine learning techniques, like SVM. Figure 5 shows this generic workflow, which should be instantiated for a specific species. 

We note that this workflow can be instantiated, according to the input data, which allows its use to other organisms. We present in this paper two examples of these instantiations, as case studies.

## 3. Results

### 3.1. Case Study 1: Sugarcane

The *Saccharum officinarum* (sugar cane) is a plant that contributes to approximately 70% of the sugar in the world. It is also used to produce paper and for feeding animals. No sugarcane genome is publicly available in biological databases, and no information about its lincRNAs is known. LincRNA prediction can help the understanding of possible roles they play on sugarcane. Thiebaut et al. [39] sequenced the sugarcane transcripts that were used to predict lincRNAs (raw input).

The sugarcane-specific SVM model used data of phylogenetically close organisms. Particularly, 2000 *Oryza sativa* (rice) and *Zea mays* (maize) lncRNAs were obtained at CantataDB [40], as well as 2000 PCTs annotated with BLAST [38], were identified among the sugarcane transcripts. In the training phase, 1600 lncRNAs and 1600 PCTs were used while, in the testing phase, 400 transcripts of each set were used. Using ORF length, ORF proportion, and 10 nucleotide pattern frequencies (AA, AT, CA, CC, CG, GA, GC, GG, TG and TT) as features, and obtaining optimal parameters *C* and *gamma* with a grid search [41], alongside 10-fold cross-validation, a SVM model was built with 82.8% of accuracy. 

**Figure 6:**
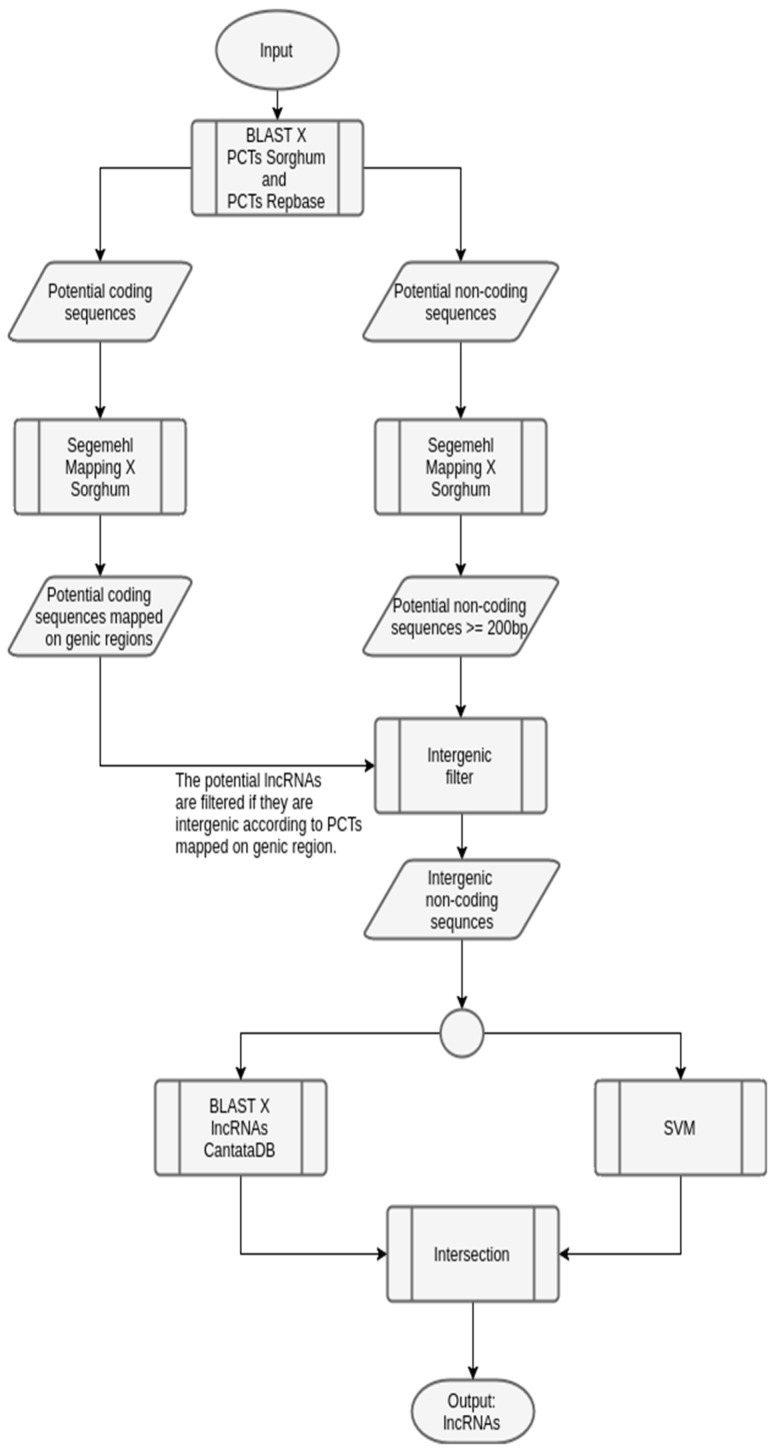
Workflow to predict lincRNAs in sugarcane, instantiated from the generic one. The input sugarcane transcripts are used in both BLAST and mapping phases, similarly to the generic workflow. Next, transcripts with lengths greater than, or equal to, 200 bp are extracted. Following, a filter was used to choose only the intergenic sequences. Here, since the sugarcane reference organism is not available, sorghum was used instead. The verification of a potential lincRNA was done considering the coding genes of sorghum. These sequences were submitted to SVM prediction and BLAST against a lncRNA database (extracted from CantataDB [40]). The output is the list of potential lincRNAs.

As sugarcane genome is not public available, data of *Sorghum bicolor* (sorghum) obtained at PlantGDB [42] was used in the mapping phase, performed with segemehl [43]. We also used data extracted from Repbase [44] in the BLAST phase, to annotate PCTs. Figure 6 shows this specific workflow.

This workflow allowed obtaining the results shown in Table 1.

We also found the differential expression of the lincRNAs predicted by the workflow in four sugarcane libraries: two treated with *Acidovorax avenae* ssp. *avenae*, the causal agent of the red stripe disease, and two control libraries. The 67 lincRNAs predicted by the workflow were mapped on these libraries, to obtain both the coverage and differential expression of each lincRNA. In total, 46 of the 67 predicted lincRNAs were differentially expressed when comparing the transcripts of the sugarcane with red stripe disease to the transcripts of the control sugarcane. 

### 3.2. Case Study 2: Maize

*Zea mays* (maize) is also a widely consumed and known plant, due to its extensive use as food for humans and other animals, for ethanol production, thickeners, and adhesive materials, and for oil production. LincRNAs for maize are not known, although some transcripts are classified as lncRNAs.

A work with maize and its treatment with *Azospirillum brasilense* and *Herbaspirillum seropedicae* is in development at the Federal University of Rio de Janeiro (UFRJ). Eight libraries from total RNA extracted from maize plants inoculated with diazotrophic bacteria were sequenced. The obtained data from the libraries were labeled as follows: id 29 and 30, libraries treated with *A. brasilense*; id 31 and 32, control; id 10 and 12, treated with *H. seropedicae*; and id 9 and 11, control.

For the SVM model, we used 4000 maize lncRNAs obtained at CantataDB [24], and 4000 PCTs obtained at Ensembl [45], using 3523 lncRNAs and 3523 PCTs for training and 477 of each set for testing. The features—ORF length, ORF proportion, and 10 nucleotide pattern frequencies (AA, AC, CA, CC, CCC, CG, GA, GC, GG and TG)—together with optimal parameters *C* and *gamma* obtained with grid search and also 10-fold cross-validation allowed building of an SVM model that achieved 99.24% of accuracy.

**Figure 7:**
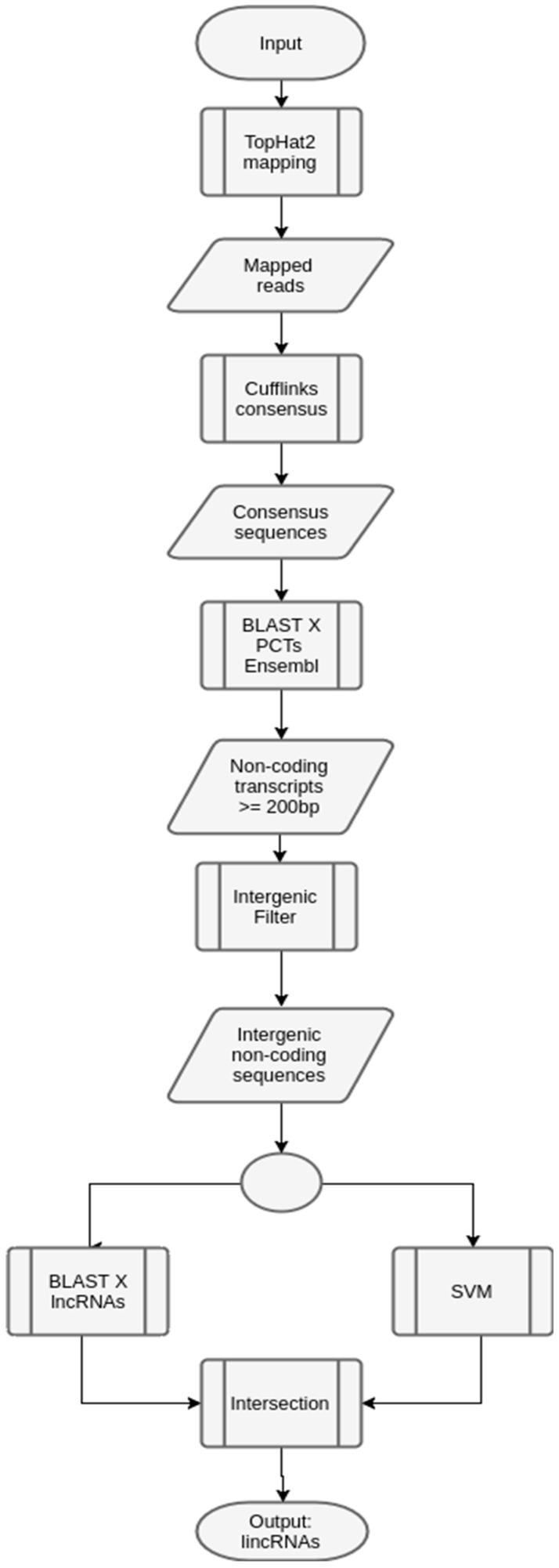
Workflow to predict lincRNAs in maize. The input maize transcripts were processed in a mapping phase, using Tophat [46], together with a phase to build the consensus sequences, using Cufflinks [47]. These two steps are not in the generic workflow. In this case, the reference genome was maize, since it is available at Ensembl [45] (*Zea mays* assembly B73 Refgen_v4). After obtaining the consensus sequences, the next phases are the same as the ones described in the generic workflow.

In this case, since the input data (transcripts) produced with the Illumina HiSeq (raw input), we have included two extra phases in the workflow: (1) mapping in the reference genome, using TopHat [46]; and (2) constructing consensus sequences using Cufflinks [47]. Figure 7 shows the workflow.

The workflow allowed to obtain the results are shown in Table 2 and Table 3. 

As we can see in Table 2 and Table 3, the output of the workflow (potential lincRNAs) for the libraries of each group (*A. brasilense* and *H. seropedicae*) have similar numbers of transcripts as the output. This could indicate that the different transcripts found in the control libraries, and the treated ones, may have a role in response to the inoculation with the endophytic diazotrophic bacteria. To verify whether there is a subset of transcripts regulated by the presence of both bacteria, we analyzed if there are subsets of transcripts common to the different treatments. 

To make this analysis, we investigated the differential expression of the predicted lincRNAs. Basically, we used BLAST to find the differential expression as explained next. First, we built a database containing the predicted lincRNAs of all the libraries (id 9, id 10, id 11, id 12, id 29, id 30, id 31, and id 32). Then, we executed BLAST with each lincRNA of each library as a query against the built database. After comparing the lincRNAs among themselves, we characterized those differentially expressed as follows: For each set of libraries (id 9 to id 12, id 29 to id 32, all the eight), a matrix was created with the sequences (predicted lincRNAs) in the rows, and the libraries in the columns. The content of one row and one column is YES (or NO), if the sequence in this row is similar (or not) to a sequence of the library in this column. Two sequences are considered similar if their BLAST e-value is less than or equal to 10^-12^. The differential expression was obtained from the combination of YES and NO in one row: at least one YES in the treated libraries and NO in the control libraries, and vice-versa. For example, taking the set of libraries id 9 to id 12, a sequence (predicted lincRNA) from library id 10 also found in library id 12 (both treated with *H. seropedicae*), and not found in libraries id 9 and id 11 (control libraries), indicates that this sequence is differentially expressed. 

Table 4 shows the differential expression of the lincRNAs predicted by the workflow, with three sets of libraries as input. These results show that while there is also a subset of transcripts commonly regulated by both bacteria, there are also transcripts that are independently regulated by each microorganism. These results are not unexpected because while both *H. seropedicae* and *A. brasilense* are diazotrophic bacteria, their growing habits are different, *H. seropedicae* is mostly endophytic and *A. brasilense* grows mostly on the surface of the roots. Additional functional analysis of the different subsets of lincRNAs may help us to understand their biological functions in the interaction with diazotrophic bacteria. Interestingly, similar observations have been made by our group analyzing the mRNA-encoding proteins in the same mRNAseq libraries. 

## 4. Conclusions

In this work, we first proposed a generic workflow to predict lincRNAs in plants that include a machine learning step based on SVM. This generic workflow can be instantiated according to each organism of interest. In order to test this workflow, we performed two case studies, for *Saccharum officinarum* (sugar cane) and *Zea mays* (maize).

Regarding sugarcane, we found putative 67 lincRNAs, one of them being tested in the laboratory [39]. Additionally, we investigated lincRNAs differentially expressed in libraries treated with *A. avenae* ssp. *avenae*, the causal agent of the red stripe disease, and two control libraries. In total, 46 of the 67 predicted lincRNAs were differentially expressed, when comparing the sugarcanes with red stripe disease with controls. 

Regarding maize, we worked with transcripts obtained from Illumina HiSeq, noting that eight libraries were produced, two treated with *Herbaspirillum seropedicae* and *Azospirillum brasilense,* respectively, while the other four were controls. In this case, our SVM model exhibited an excellent accuracy of 99%. Additionally, in this case, we investigated differentially-expressed lincRNAs comparing the treated libraries with the control ones. 

In both case studies, the number of sequences predicted as lincRNAs with BLAST and SVM were very different. This indicates that our SVM model is not so stringent as BLAST. We also note that, in both case studies, the output of the workflow shows that the number of lincRNAs is much lower when compared to the number of the input sequences, and we think that they are very likely to be lincRNAs.

Another interesting aspect is that building an SVM model specific to each organism improves accuracy. We believe that this is more efficient to predict lincRNAs than building a generic SVM model, since it considers the specificities of an organism of interest. Furthermore, a workflow that uses machine learning and other bioinformatics tools can solve the lack of stringency of the SVM model. It also improves the performance of BLAST, while assuring a more reliable lincRNA prediction in plants.

Nevertheless, these in silico predictions should be tested to confirm if they really are lincRNAs. 

## Figures and Tables

**Table 1:** Predicted lincRNAs in sugarcane.

Library	Number of Sequences
Raw Input	168,767
Filtered input (transcripts not annotated as PCTs)	63,389
Transcripts mapped on sorghum gene regions	9488
Non-coding transcripts mapped on sorghum	4425
Non-coding transcripts mapped on intergenic region (using the sorghum genome as reference)	2432
LincRNAs predicted by the SVM model	1689
LincRNAs annotated by BLAST	97
LincRNAs annotated by BLAST and predicted by SVM model	67

PCT: protein coding transcripts; lincRNA: long intergenic non-coding RNA; SVM: support vector machine.

**Table 2:** Predicted lincRNAs in maize, libraries treated with *Herbaspirillum seropedicae*.

Library	id 9 (Control)	id 10	id 11 (Control)	id 12
Raw input	7,158,821	5,032,782	8,277,629	6,327,933
Mapped Reads	4,528,477	3,161,660	8,321,623	3,662,784
Consensus sequences	156,565	155,472	157,562	155,578
Non-coding transcripts ≥ 200 bp	2988	2731	3168	2792
Intergenic non-coding transcripts ≥ 200 bp	2776	2536	2938	2592
lincRNAs predicted by SVM	2743	2512	2904	2567
lincRNAs annotated by BLAST	513	420	543	446
lincRNAs annotated by BLAST and predicted by SVM	507	418	539	444

**Table 3:** Predicted lincRNAs in maize, libraries treated with *Azospirillum brasilense*.

Library	id 29	id 30	id 31 (Control)	id 32 (Control)
Raw input	17,555,365	15,322,340	17,149,619	15,497,154
Mapped Reads	11,904,702	10,246,454	11,847,394	10,745,811
Consensus sequences	161,476	160,962	162,208	161,167
Non-coding transcripts ≥ 200 bp	3985	3760	4405	3999
Intergenic non-coding transcripts ≥ 200 bp	3554	3381	3866	3534
lincRNAs predicted by SVM	3494	3330	3814	3486
lincRNAs annotated by BLAST	737	692	877	765
lincRNAs annotated by BLAST and predicted by SVM	732	687	870	759

**Table 4:** Differential expression of the lincRNAs in maize, two treated with *H. seropedicae* and two control libraries, and two treated with *A. brasilense* and two more control libraries. All of those lincRNAs occurring in at least one of the analyzed libraries and not occurring in the others (treated and control) are considered as differentially expressed.

Set of Libraries	Number of LincRNAs Differentially Expressed
*H. seropedicae* (libraries id 9, id 10, id 11 and id 12)	267
*A. brasilense* (libraries id 29, id 30, id 31 and id 32)	476
*H. seropedicae* and *A. brasilense* (all the libraries: id 9, id 10, id 11, id 12, id 29, id 30, id 31 and id 32)	1164

## Supplementary Materials

Sequences of the transcripts used in the two case studies are available at annex.
